# Preparation and Characterization of Chitosan/Starch Nanocomposites Loaded with Ampicillin to Enhance Antibacterial Activity against *Escherichia coli*

**DOI:** 10.3390/polym16182647

**Published:** 2024-09-19

**Authors:** Vinh Nghi Nguyen, San-Lang Wang, Thi Huyen Nguyen, Van Bon Nguyen, Manh Dung Doan, Anh Dzung Nguyen

**Affiliations:** 1 Ninh Thuan Hospital, Phan Rang-Thap Cham City 59000, Vietnam; nguyenvinhnghi0607@gmail.com; 2 Institute of Biotechnology and Environment, Tay Nguyen University, Buon Ma Thuot 630000, Vietnam; nthuyen@ttn.edu.vn (T.H.N.); nvbon@ttn.edu.vn (V.B.N.); dmdung@ttn.edu.vn (M.D.D.); 3 Department of Chemistry, Tamkang University, New Taipei City 25137, Taiwan; 4 Life Science Development Center, Tamkang University, New Taipei City 25137, Taiwan

**Keywords:** chitosan, starch, nanocomposites, ampicillin, *E. coli*, drug delivery

## Abstract

Chitosan/starch nanocomposites loaded with ampicillin were prepared using the spray-drying method by mixing various ratios of chitosan and starch. The morphology of chitosan/starch nanoparticles was studied using a scanning electron microscope (SEM), and the zeta potential value and size distribution were determined by a Nanoparticle Analyzer. The results show that the chitosan/starch nanocomposites have a spherical shape, smooth surface, and stable structure. Nanoparticle size distribution ranged from 100 to 600 nm, and the average particle size ranged from 300 to 400 nm, depending on the ratio between chitosan and starch. The higher the ratio of starch in the copolymer, the smaller the particle size. Zeta potential values of the nanocomposite were very high, ranging from +54.4 mV to +80.3 mV, and decreased from 63.2 down to +37.3 when loading with ampicillin. The chitosan/starch nanocomposites were also characterized by FT-IR to determine the content of polymers and ampicillin in the nanocomposites. The release kinetics of ampicillin from the nanocomposites were determined in vitro using an HPLC profile for 24 h. The loading efficiency (LE) of ampicillin into chitosan/starch nanoparticles ranged from 75.3 to 77.3%. Ampicillin-loaded chitosan/starch nanocomposites were investigated for their antibacterial activity against antibiotic-resistant *Escherichia coli* in vitro. The results demonstrate that the antibacterial effectiveness of nanochitosan/starch loading with ampicillin against *E.coli* was 95.41%, higher than the 91.40% effectiveness of ampicillin at the same concentration of 5.0 µg/mL after 24 h of treatment. These results suggest that chitosan/starch nanocomposites are potential nanomaterials for antibiotic drug delivery in the pharmaceutical field.

## 1. Introduction

*Escherichia coli* causes diarrhea, sepsis, and other clinical symptoms; it is one of the main enteric pathogens affecting human and animal health. Currently, nearly all bacteria that cause serious illnesses are resistant to antibiotics [1]. According to the WHO (2018), antibiotic resistance in pathogenic bacteria (AMR: antimicrobial resistance) is spreading and poses a serious challenge to the healthcare system [2]. Ampicillin (AMP), a semisynthetic beta-lactam antibiotic, is widely used to treat *E. coli* infections in humans and livestock, but the rate of resistance has increased rapidly. The ampicillin resistance rate of *E. coli* reaches up to 82.7% [3,4].

To date, to fight drug-resistant *E. coli*, a fairly effective solution has involved the use of nanomaterials to carry antibiotics. These nanomaterials operate through various mechanisms, such as degrading cell walls; decomposing and preventing biofilm formation; releasing activated oxygen (ROS) that destroys cell membranes; destroying DNA, and preventing protein synthesis [5,6,7,8]. Chitosan nanoparticles, based on the linear polysaccharide chitosan, have a larger contact surface area and positive charge than regular chitosan, so they have higher antibacterial activity [9]. Recently, studies have demonstrated that chitosan nanoparticles are very potent drug carriers and delivery nanomaterials. They can protect drugs from being broken down by enzymes in the intestinal tract. Due to their small size, they can penetrate deeply into the body, delivering drugs to the correct target tissues and cells to improve treatment effectiveness. Many studies have used chitosan to carry antibiotics, such as amoxicillin, ciprofloxacin, chlortetracycline, and gentamycin, enhancing efficiency and fighting drug resistance in pathogenic bacteria [10,11,12,13,14,15]. However, chitosan nanoparticles are limited by their high cost and instability in acidic pH conditions, which restricts their application in the nanoparticle pharmaceutical field. Due to these limitations, chitosan was incorporated with other polymers such as alginate, cellulose, and starch [16,17,18].

Meanwhile, starch, as a natural branched polysaccharide, is a safe, low-cost, and biocompatible polymer that is widely applied in the food and pharmaceutical fields [19,20]. Starch has certain limitations due to its poor functional properties, negative charge, and digestion by pancreatic enzymes in the small intestine [21,22]. Therefore, starch may be blended with chitosan or synthetic polymer to improve its physical properties for pharmaceutical applications [23,24].

This study focuses on the preparation of chitosan/starch nanocomposites to address the above-mentioned advantages and limitations of the individual polymers and to improve their biophysical properties. Therefore, this study aims to find new chitosan/starch nanocomposites (prepared by spray-drying) that are nanometer-sized and exhibit high positive zeta potential value, high loading efficiency, low cost, and stability in acidic environments and the small intestine. These properties are important in the fight against antibiotic-resistant bacteria.

## 2. Materials and Methods

### 2.1. Materials

Chitosan was obtained from Merck Sigma Chemical Co. (St. Louis, MO, USA) with Mw 500–750 kDa, DD > 75%. *E. coli* strain was provided by the Pasteur Institute, Ho Chi Minh City, Vietnam. Ampicillin sulfate was purchased from Sigma Aldrich, USA. Soluble starch A.R and Nutrient Broth were purchased from HiMedia, (Mumbai, Maharashtra, India).

### 2.2. Methods

*Method of loading ampicillin into chitosan/starch nanocomposites.*

A total of 0.1 g of chitosan was dissolved into 100 mL of 0.1 M acetic acid using a magnetic stirrer. The starch was gelatinized to form a gel at a concentration of 0.1% (*w*/*v*) in deionized water at a temperature of 100 °C. The chitosan solution was mixed with starch gel at a ratio (*w*/*w*) between chitosan (C) and starch (S), such as 1:0 (C100); 1:1 (C1S1); 1:2 (C1S2),1:3 (C1S3), and 0:1 (S100), and stirred well for 60 min. Then, 5 mg of ampicillin was added to the 100 mL chitosan/starch gel mixture to reach a final concentration of 50 µg/mL. Chitosan/starch nanocomposites loaded with ampicillin were prepared using a spray dryer (Nano B90, Labortechnik AG Meierseggstrasse, Buchi, Flawil, Switzerland), according to the following parameters: input temperature—120 °C; output temperature—80 °C; spray flow rate—120 mL/h; compressed air pressure—1.2 m^3^/min; and nozzle size—5.0 µm. The parameters followed the process described by La et al., 2014 [12]. The chitosan/starch nanocomposite morphology was characterized by SEM (Phenom ProX, Thermo Scientific, Waltham, MA, USA) at 10 kV. The size, zeta potential, and size distribution of the nanocomposites were measured by a Nanoparticle Analyzer (SZ 100, Horiba, Kyoto, Japan). The physicochemical properties of chitosan/starch nanocomposites loaded with ampicillin were also determined by FT-IR (Alpha, Brucker, Billerica, MA, USA) with a range of 500–4000 cm^−1^ at a resolution of 16 cm^−1^ within 32 scans.

*In vitro-released ampicillin kinetics of chitosan/starch nanocomposites.*

The release kinetics of ampicillin from chitosan/starch nanocomposites were conducted with five samples: chitosan nanoparticles (C100), starch nanoparticles (S100), C1S1, C1S2, and C1S3-loaded ampicillin.

The loading efficacy (LE%) was determined by centrifugation and by processing data from kinetic studies [25] as follows: 50 µg of nanocomposite samples were suspended in 500 µL of PBS (pH = 7.0) and subjected to high-speed centrifugation (14,000 rpm at 5 °C). The supernatant containing the unencapsulated ampicillin was isolated, and the nanocomposite loaded with ampicillin was resuspended in ampicillin-free PBS to test the release kinetics at room temperature for 24 h. The ampicillin content was assayed using an HPLC (UHPLC-Thermo 3000) with a Hypersil GOLD PFP (150 mm × 2.1 mm, 3 µm, Thermo Scientific, Waltham, MA, USA) at 25 °C. The mobile phase was MeOH/ACN/HCOOH = 30/40/30. The analysis condition was set at a flow rate of 0.2 mL/min and a detecting wavelength of 270 nm. The linear regression equation of ampicillin and the HPLC profiles of ampicillin standard and ampicillin loaded into chitosan/starch nanocomposites are presented in Figures S1–S8. These data are used to calculate the percent loading (LE%) and release kinetics.

$$LE=(\frac{Qt-Qs}{Qt})\times 100$$

where *LE*, *Qt*, and *Qs* correspond to the loading efficiency, the initial total amount of ampicillin, and the amount of unencapsulated ampicillin, respectively.

*Method for evaluating the antibacterial activity of chitosan/starch nanocomposites loaded with ampicillin in vitro.*

Nutrient broth was autoclaved at 121 °C for 20 min; 10 mL of nutrient broth (after sterilization) was supplemented with nanochitosan/starch nanocomposites—C100; C1S1; C1S2; C1S3, and S100—loaded with ampicillin to reach the final concentrations of 0; 0.5; 1.0; 2.5; 5.0; 7.5 μg/mL of ampicillin. Then, 50 μL of *E. coli* suspension (10^6^ CFU/mL) was added to the medium and cultured at 30 °C, 120 rpm, for 48 h. Each experimental batch was conducted in triplicate. The antibacterial activity was determined by measuring turbidity at 620 nm using a UV-Vis spectrophotometer (Jasco, Japan) at 0; 3; 6; 9; 12; 18; 24; 36, and 48 h. The antibacterial activity was calculated according to the formula described by Asadpoor et al., 2021 [26] as follows:

I (%) = (B − K)/B × 100%

where I denotes the relative inhibition rate (%); B denotes the mean of absorbent values at OD620 nm in the control; and K denotes the mean of absorbent values at OD620 nm in the treatments.

*Data analysis*.

The one-way ANOVA combined with an LSD test (*p* < 0.05) was used to assess the differences among the treatments. The data are graphically presented by mean ± standard deviation (SD).

## 3. Results and Discussion

### 3.1. Preparation and the Properties of Chitosan/Starch Nanocomposites Loaded with Ampicillin

To reduce the input costs and increase the application potential of chitosan, in this work, chitosan was combined with starch, which is a safe, inexpensive biopolymer by nature. The properties of chitosan/starch composite nanoparticles (Table 1) show that the nanoparticle size decreased gradually as the ratio of chitosan in the nanocomposites decreased, from 816.9 nm (100% chitosan C100) to 372.4 nm in the formula with a 50% chitosan:50% starch (C1S1) ratio. In the formula containing 100% starch (S100), the nanoparticle size mean was 133.2 nm. Nguyen et al. (2017) reported that chitosan nanoparticles prepared by spray-drying had a distribution size ranging from 300 to 750 nm, zeta potential values of +40 mV to +50 mV, and a mean size of 420 nm [9]. Compared to this study, the C1S3 sample of chitosan/starch nanoparticles had a smaller mean size of 190 nm, which is smaller than what was reported in Nguyen’s work. La et al. (2014) produced chitosan nanoparticles loaded with amoxicillin via spray-drying. The results show that the mean size of chitosan nanoparticles ranged from 100 to 400 nm, and zeta potential values ranged from +39 mV to +45 mV. Chitosan nanoparticles were also used as nanocarriers for the delivery of amoxicillin to fight *S. aureus*. The inhibitory rate of amoxicillin loaded onto chitosan nanoparticles was higher than that of amoxicillin in the same concentration. The study also reported that the size and properties of chitosan nanoparticles depended on the molecular weight and concentration of chitosan [12]. The results shown in Table 1 also demonstrated that the mean size of the nanocomposites was higher than that of loading with ampicillin. The mean size was 372.4 nm (C1S1), which increased to 583.9 nm (C1S1 + Amp); and it was 281.0 nm (C1S2), which increased to 566.4 nm (C1S2 + Amp).

Zeta potential is an important physical property of nanoparticle colloids as it affects their stability. The stability of nanoparticles in the solution can be determined based on the zeta potential value. The nanoparticles that are stable and uncoagulated in the solutions possess zeta potential values greater than ± 30 mV [27]. The results (Table 1, Figure S9) indicate that the zeta potential values of the chitosan/starch nanocomposites range from +54.4 mV to + 80.3 mV, depending on the ratio of starch in the nanocomposites. The higher the ratio of starch in the nanocomposites, the lower the zeta potential values. The zeta potential (C1S1) is +80.3 mV, decreasing to +71.4 mV (C1S2) and + 54.4 mV (C1S3). This causes the zeta potential value of starch (S100) to be negatively charged (−19.7 mV). Similarly, the loading of ampicillin (negative charge) leads to a decrease in the zeta potential values of nanocomposites, such as C1S1 (+80.3 mV), C1S1 + Amp (+63.2 mV), C1S3 (+54.4 mV), and C1S3 + Amp (+37.3 mV). These results indicate that the presence of ampicillin at a concentration of 5 microgram/mL in the loaded nanocomposites reduced the zeta potential values by 14.1% to 31.4% compared to the unloaded nanocomposites.

Additionally, there have been numerous studies reporting the use of chitosan nanoparticles as nanocarriers for the delivery of antibiotics. However, there have been no reports on the preparation of chitosan/starch nanocomposites by spray-drying or their use as nanocarriers for antibiotic delivery, until now. Hussein-Al-Ali et al. (2014) prepared nanocomposites of ampicillin-conjugated chitosan and iron oxide nanoparticles. The results showed that the zeta potential value was low (+14.4 mV), with an average size of 40.0 nm [28]. Abdel-Hakeem et al. (2022) reported that gentamycin was encapsulated in chitosan nanoparticles via ionic gelation with tripolyphosphate. The chitosan nanoparticles had mean sizes in the range of 150–250 nm and a zeta potential value of +30 mV [29]. Other works reported that antibiotics loaded into nanocarriers enhanced antibacterial activity compared to free antibiotics [30,31].

Fan et al. (2022) loaded two cephalosporin antibiotics and β-lactamase inhibitors (CNAIs) into chitosan nanoparticles, engineered using a water/oil/water microemulsion method. The particle sizes ranged from 200 to 250 nm, and the loading efficiencies of cefotaxime and ceftiofur were approximately 70% and 60%, respectively. The results also demonstrated that CNAIs showed higher antimicrobial activity compared to naive chitosan nanoparticles and to the combination of cephalosporin antibiotics with β-lactamase inhibitors [32].

**Figure 1:**
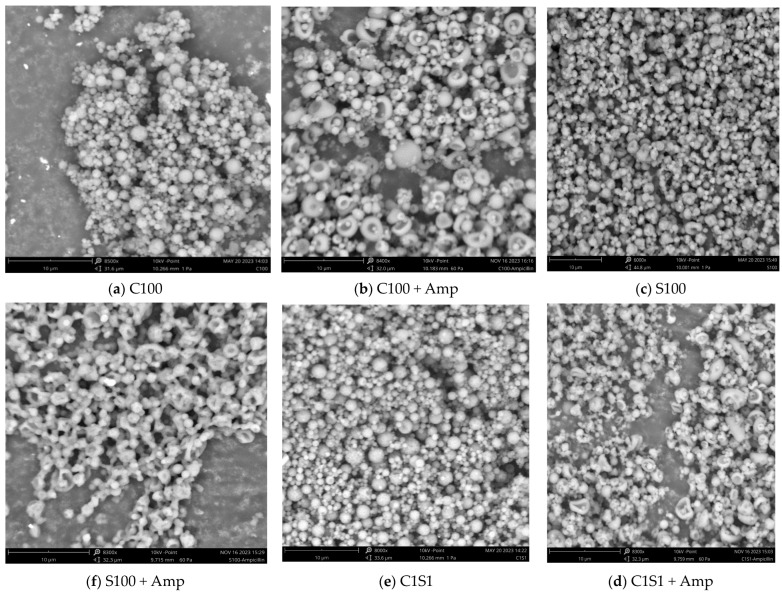

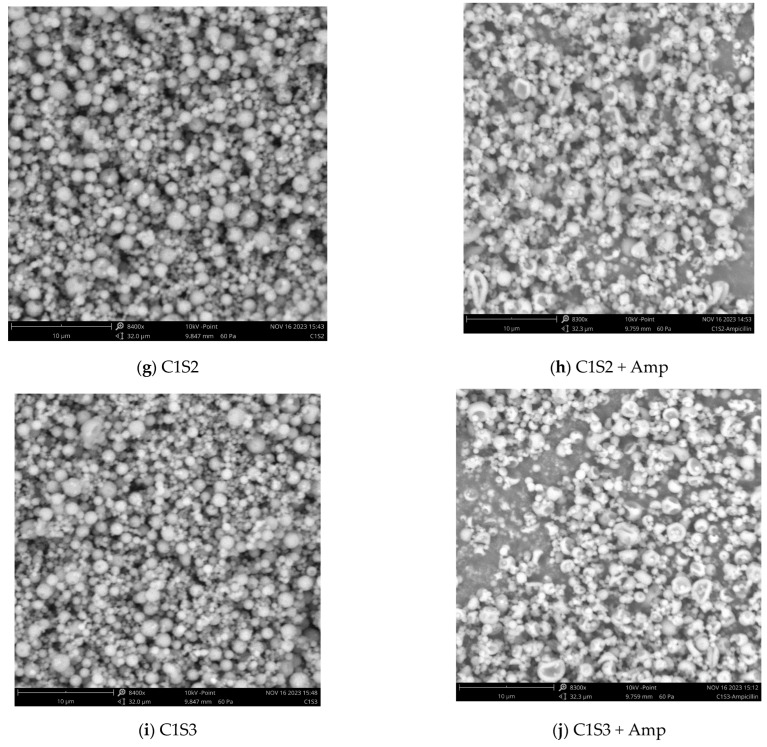
SEM of chitosan/starch nanocomposites and the nanocomposites loaded with ampicillin (taken by SEM Phenom ProX, Thermo Scientific, Waltham, MA, USA; 8000×; 10 kV; bar 10 μm).

The morphologies of the chitosan/starch nanocomposites and chitosan/starch nanocomposites loaded with ampicillin were characterized by SEM (Figure 1). The structure and surface of the chitosan/starch nanocomposites had a solid, spherical, and uniform structure, with a smooth surface. The spherical structure provides the largest volume and surface area, which is advantageous in encapsulating and releasing the drug better than other structures. The results of SEM also demonstrated that the morphology of the nanoparticles—after loading with ampicillin—appeared concave on the surface. The reason may be that ampicillin has a negative charge that changes the nanoparticle charge, leading to a change in surface tension and creating a concave surface. This morphology appeared to occur only in large-sized nanoparticles (Figure 1b,d,f,h,j).

**Figure 2:**
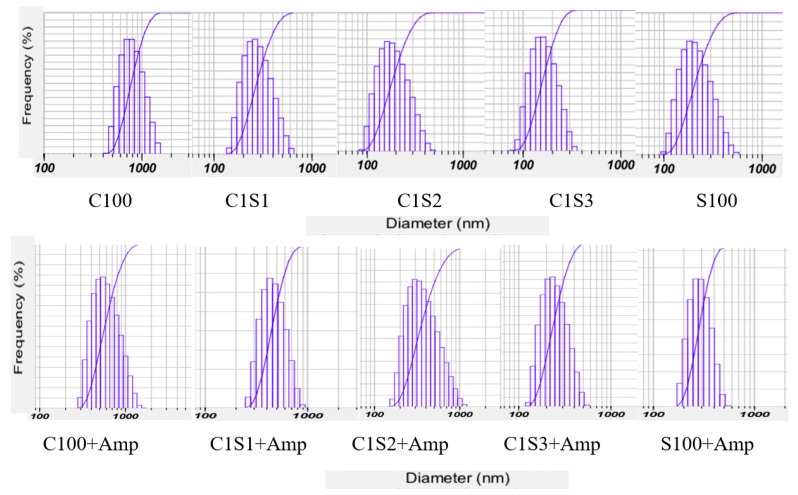
The size distribution of chitosan/starch nanocomposites and chitosan/starch nanocomposites loaded with ampicillin by a Nanoparticle Analyzer (SZ 100, Horiba, Kyoto, Japan).

The size distribution indicates the uniformity of the nanoparticle sizes. The narrower the particle size distribution, the more uniform the particle size. The results (Figure 2) show that the size distribution of all the samples followed the Gause distribution. The larger the starch ratio in the copolymer, the smaller the particle size and the narrower the size distribution. The results show that samples had a distribution of 400–1600 (C100); 90–500 (S100); 140–600 nm (C1S1); 100–500 nm (C1S2); and C1S3 from 90 to 400 nm (Figure 2). The size distribution of nanoparticles was larger when loading with ampicillin (Table 1, Figure 2).

**Figure 3:**
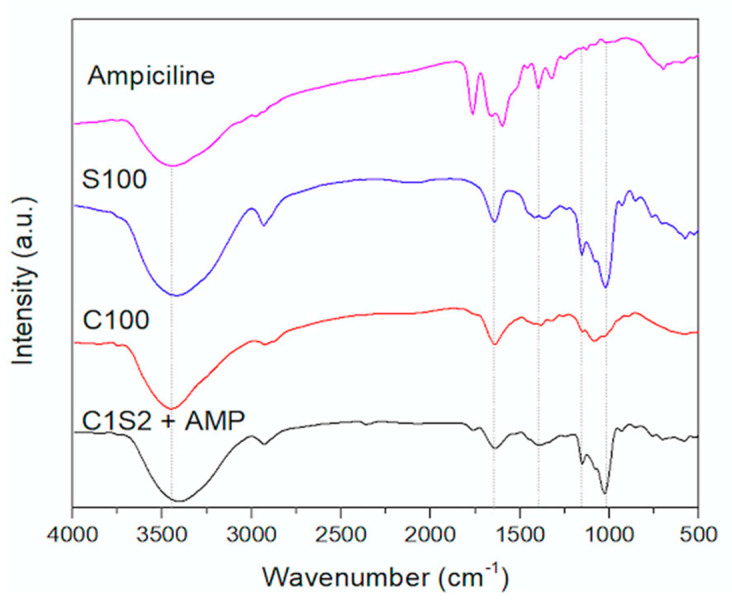
FTIR of ampicillin loaded into chitosan/starch nanocomposites (FTIR Alpha, Brucker, Billerica, MA, USA) with a range of 500–4000 cm^−1^ at a resolution of 16 cm^−1^ within 32 scans).

FTIR analysis of ampicillin loaded into chitosan/starch nanocomposites: The FTIR analysis of ampicillin (Amp) is shown in Figure 3, demonstrating that a peak at 3444 cm^−1^ was associated with N-H stretching of the primary amine and the secondary amide of the beta-lactam ring, as well as O-H stretching of the carboxylic group present in ampicillin. The peaks around 2900 cm^−1^ were attributable to the symmetrical and asymmetrical stretching of –C–H. The peak at 1765 cm^−1^ was the most characteristic peak of ampicillin, indicating stretching of the carbonyl (C=O) of the β-lactam ring. Peaks between 1660 cm^−1^ and 1600 cm^−1^ were associated with the bending of N-H_2_, the primary amine group of ampicillin. Eventually, the peaks in the region between 1500 cm^−1^ and 1300 cm^−1^ were associated with the C=C stretching of the aromatic ring and the N–H bending of the secondary amide, whereas the peak at 1251 cm^−1^ was due to the C–N stretching of the primary amine [33,34,35].

The FTIR spectrum of starch (S100) presented in Figure 2 shows that the peaks at 3419 cm^−1^, 2931 cm^−1^, 1154 cm^−1^, 1079 cm^−1^, and 1022 cm^−1^ in the spectrum correspond to -OH stretching, -C-H symmetric vibration, C-O-C, and the C-O functional group, respectively. In addition, the characteristic C-O-C ring vibration on starch leads to an absorbance peak at around 930 cm^−1^. The C-O bending associated with the OH group would cause an absorbance peak at around 1642 cm^−1^ [36,37].

The FTIR spectrum of the chitosan (C100) sample in Figure 3 indicates that chitosan exhibited characteristic peaks for vibrations of the bonds in chitosan as follows: The peak at 3449 cm^−1^ was due to the -OH stretching. The peak at 2924 cm^−1^ was typical for the C–H symmetric vibration. The peak at 1641 cm^−1^ characterized the N–H bending while the peak at 1085 cm^−1^ corresponded to C-O stretching [16,28,31,38].

The FTIR spectrum of chitosan/starch nanocomposites loaded with ampicillin (C1S2 + AMP) indicate that all the characteristic peaks were associated with ampicillin, starch, and chitosan, which were ingredients in the composite; this included peaks at around 3450 cm^−1^ (-NH stretching of amide in the ampicillin; and -OH stretching of ampicillin, starch, and chitosan) and around 2900 cm^−1^ (-CH stretching of ampicillin, starch, and chitosan). The peak at 1765 cm^−1^ indicated the stretching of the carbonyl (C=O) of the β-lactam ring (ampicillin); 1640 cm^−1^ corresponded to -NH bending of chitosan and the C-O bending of starch in the nanocomposites (C1S2 + AMP). Therefore, the FTIR spectrum of the nanocomposite-loaded ampicillin (C1S2 + AMP) demonstrated that the ingredients of the nanocomposite-loaded ampicillin consisted of ampicillin, starch, and chitosan.

### 3.2. In Vitro Loading Efficiency and Release Kinetics of Ampicillin

**Figure 4:**
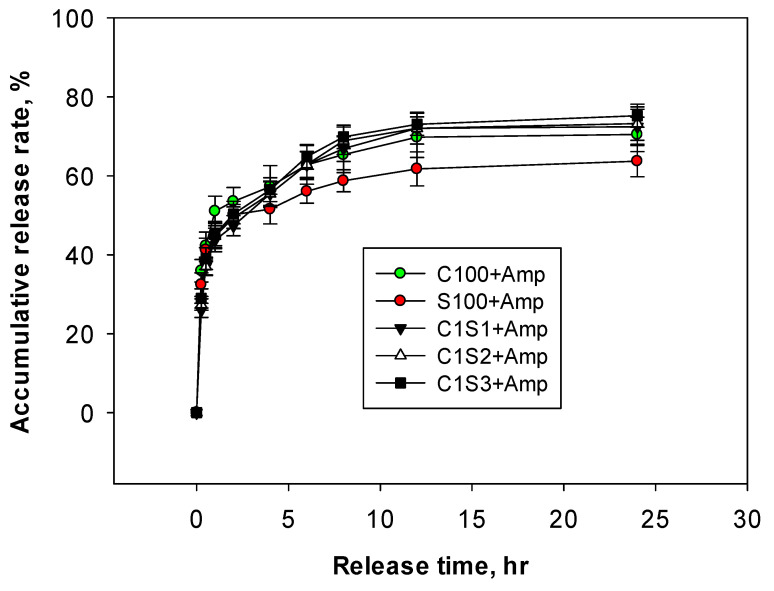
The release kinetics of ampicillin from the nanocomposites (in phosphate buffer solution at pH = 7.0, 25 °C).

The results shown in Table 1 and Figure 4 indicate that the loading efficiency (LE) of the chitosan/starch nanocomposites ranged from 75.3 to 77.3%. This is significantly higher compared to the loading efficiencies of chitosan nanoparticles (C100) at 65.8% and starch nanoparticles (S100) at 59.9%. This study indicates that the matrix structure formed by the composites of chitosan and starch facilitates better encapsulation of ampicillin compared to individual chitosan and starch nanoparticles.

The release kinetics of ampicillin from chitosan/starch nanocomposites, as illustrated in Figure 4 and Figures S1–S8, demonstrating a rapid initial release phase followed by a slower release over time. The data indicate that approximately 50% of the ampicillin was released within the first hour. After the first hour, the release rate decreased, with the following percentages of ampicillin released at 12 h: approximately 70% for the C100 + Amp formulation, and between 72.4% to 75.2% for the C1S1 + Amp, C1S2 + Amp, and C1S3 + Amp formulations. The S100 + Amp formulation shows a slightly lower release rate at 63.7%. Chitosan is a linear polysaccharide. With no matrix structure, the chitosan nanoparticles (C100) had an ampicillin LE% value of 65.5%, which is lower than those of the other nanocomposites (Table 1). This is also the main reason why the release kinetics of ampicillin from chitosan nanoparticles are quick in the initial phase (Figure 4). Starch, being a negatively charged branched polysaccharide, blends with positively charged chitosan to form a firm, stable, and matrix-structured composite. In addition, since starch and ampicillin have the same negative charge, the loading efficiency of starch (S100) was only 59.9%, which is lower than the others (Table 1). After 12 h, the release rates stabilize, indicating that the majority of the drug has been released and that the release kinetics are influenced by the chitosan/starch ratio within the nanocomposites.

In comparison, another study investigated the release of ampicillin from a nanocomposite incorporating iron oxide magnetic nanoparticles. In that study, the release profile was noted as follows: around 50% of ampicillin was released within 0.5 h, 67% within 1 h, and nearly 100% within 6 h [28]. Additionally, Nairi et al. (2017) explored the adsorption and release of ampicillin from ordered mesoporous silica (OMS). The results indicated that 56% and 42% of ampicillin antibiotics were released from mesoporous silica MCM-41 and SBA-15 within the first two hours [34]. Ciprofloxacin was also loaded into chitosan nanoparticles using the ionic gel method. The obtained results revealed an average particle size of 250 nm and a zeta potential value of 38.5 mV. The release profile indicated that approximately 83% was initially released, followed by a slowed drug release (up to 24 h), similar to the results of this study [39]. Loading two cephalosporin antibiotics and β-lactamase inhibitors (CNAIs) into the chitosan nanoparticles was engineered using a water/oil/water microemulsion method. The loading efficiencies of cefotaxime and ceftiofur were approximately 70% and 60%, lower than those in this study [32].

The findings suggest that the ampicillin release profile from the chitosan/starch nanocomposites is superior compared to the C100 + Amp and S100 + Amp formulations, likely due to the physically and chemically enhanced interaction between the two polymers, and encapsulation efficiency of the drug within the nanocomposite matrix. Blending two polymers that complement each other in terms of characteristics and limitations—such as linear and matrix structures, positive and negative charges, expensive and cheap costs, and acid stability and instability—allows the formation of novel nanocomposites for drug delivery systems. Overall, the results emphasize the importance of the nanocomposite composition in encapsulating and controlling the release kinetics of ampicillin, which is critical for optimizing therapeutic efficacy and minimizing side effects in drug delivery applications.

### 3.3. Effect of Ampicillin-Loaded Chitosan/Starch Nanocomposites on Antibacterial Activity against E. coli In Vitro

**Figure 5:**
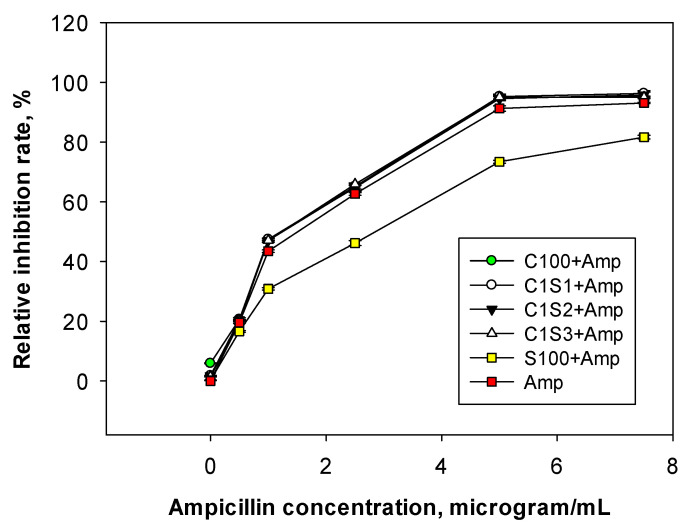
Effect of ampicillin concentration loaded into the nanocarriers on the relative inhibitory rate against *E. coli* (cultured in nutrient broth, at 30 °C, 120 rpm for 48 h).

The effect of ampicillin-loaded chitosan/starch nanocomposites on *E. coli* antibacterial activity was evaluated at concentrations ranging from 0 to 7.5 μg/mL for 12 h of incubation. The sample was then measured for turbidity at OD620 nm and the inhibitory rate was determined. This experiment aimed to identify the minimal inhibitory concentration of ampicillin loaded into the nanocarriers for the next experiments. The results presented in Figure 5 show that increasing the antibiotic concentration within the nanocomposites increased the antibacterial effect on *E. coli*. At a concentration of 1.0 μg/mL, both the chitosan/starch nanocomposites and free carrier (ampicillin) showed an inhibitory rate of approximately 50%, reaching 65–70% at a concentration of 2.5 μg/mL. At a concentration of 5.0 μg/mL, ampicillin loaded into chitosan/starch nanocomposite carriers, such as C100, C1S1, C1S2, and C1S3 + Amp, had an inhibitory rate of over 95.4%, compared to 92.2% for free ampicillin (Amp) and 74.3% of starch nanoparticles loaded with ampicillin (S100 + Amp). Ampicillin achieved a 95.0% inhibitory rate at a concentration of 7.5 μg/mL. The chitosan/starch nanocarriers loaded with ampicillin had a positive charge of +37.7 to +67.7 mV (Table 1), which helped them to absorb and go through the negatively charged bacterial cell membrane more effectively than the ampicillin and starch nanoparticles (S100 + Amp). The ampicillin was trapped in the nanocarrier and released slowly. Therefore, these properties limit the ability of bacteria to resist ampicillin antibiotics.

The results in Figure 5 also show that in ampicillin encapsulated into starch nanoparticles (S100 + Amp), the bacterial inhibitory rate against *E. coli* was lower than those of free ampicillin and ampicillin in chitosan/starch nanocomposite carriers. The reason may be that starch nanoparticles (S100) had charges of −19.7 mV and −6.3 mV (S100 + Amp) (Table 1), so the ability to access the negatively charged cell membrane was more difficult compared to the others. In addition, the structure of starch nanoparticles (S100) when carrying ampicillin exhibited a phenomenon of chaining (Figure 1d), which reduced their ability to approach the bacterial cells, resulting in a lower antibacterial effect against *E. coli* (only 70.5% at ampicillin of 5.0 μg/mL).

### 3.4. Effect of Ampicillin-Loaded Chitosan/Starch Nanocomposites and Incubation Time on E. coli Antibacterial Activity

**Figure 6:**
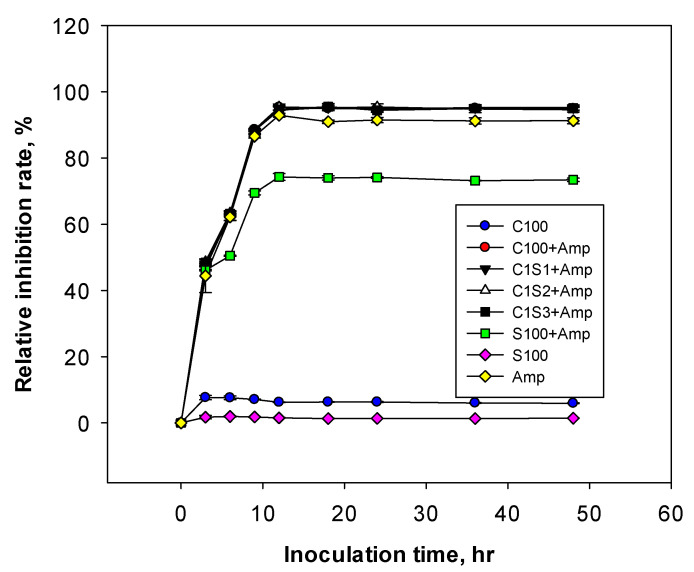
Effect of chitosan/starch nanocomposites loaded with ampicillin on antibacterial activity against *E. coli* in vitro (in nutrient broth with a concentration of 5.0 μg/mL ampicillin, at 30 °C, 120 rpm, for 48 h).

To study the antibacterial effect and the ability to inhibit drug resistance of ampicillin (5.0 μg/mL) encapsulated in chitosan/starch nanocomposites, an experiment was conducted with seven samples of nano chitosan/starch composite and incubation time of 48 h (Figure 6). The results in Figure 4 show that all samples of chitosan/starch nanocomposites, such as C100; C1S1; C1S2; C1S3; and C2S1; and C3S1 loaded with 5.0 μg/mL of ampicillin, had a high inhibitory rate of 95.0–95.41%, which is higher than only ampicillin at 92.9% and starch nanoparticles (S100) at 74.3%. In particular, the antibacterial effects of chitosan/starch nanocomposites loaded with ampicillin remained stable after 18 h, 24 h, 36 h, and 48 h of incubation. Meanwhile, the ampicillin sample decreased rapidly from 92.9% at 12 h to 90.97% at 18 h. These results demonstrate that there was an increase in the drug resistance of *E. coli,* which led to a reduction in the antibacterial activity of ampicillin. The results shown in Figure 6 indicate that the antibacterial activity of chitosan/starch nanocomposites loaded with ampicillin, such as samples C1S1 + Amp, C1S2 + Amp, C1S3 + Amp, were no different from those of the C100 + Amp. This study finds that starch may partially substitute chitosan in the nanocomposites, reducing costs and enhancing the stability, loading efficiency, and antibacterial activity of ampicillin.

Li et al. (2019) reported that *E. coli* has several ways to resist the activity of ampicillin, such as inducing beta-lactamase to degrade and inactivate ampicillin; changing membrane permeability to antibiotics; and pumping antibiotics back out of the cell membrane [3]. In this study, loading ampicillin into chitosan/starch composite nanoparticles may be able to prevent degradation by beta-lactamase in *E. coli* and decrease the pumping of ampicillin-loaded nanocomposites out of the cell membrane. In addition, nanocomposites loaded with ampicillin, which are nanometer-sized and have a large positive charge (+70 to +90 mV), increase the penetration of the nanoparticles through the cell membrane.

To date, many works have reported using nanocarriers for antibiotic delivery, and almost all results showed that the antibacterial activity of antibiotics loaded into nanocarriers is significantly higher than that of free antibiotics. However, most of these works only used chitosan nanoparticles [9,12,13,14,15,16,29,30,31,32] and starch nanoparticles [40,41,42]. A few works reported a combination of chitosan with alginate, cellulose, and magnetic materials to form nanocomposites [17,18,28,34].

## 4. Conclusions

The findings of this study demonstrate that chitosan/starch nanocomposites, prepared through spray-drying, yield uniform, spherical, and stable nanoparticles with a mean size from 300 to 500 nm. The characteristics of these nanocomposites, such as morphology, size, size distribution, and zeta potential values, are significantly influenced by the ratio of chitosan to starch in the nanocomposites. In particular, an increased starch ratio results in smaller particle sizes and lower zeta potential values. These nanocomposites show promise as effective nanocarriers for the delivery of ampicillin antibiotics. The ampicillin-loading efficiency of chitosan/starch nanocomposites C1S2 and C1S3 is higher than that of the other nanocomposites. The release profile of ampicillin from the nanocarriers is stable, with optimal chitosan to starch ratio, identified as 1:2 or 1:3 (*w*/*w*), corresponding to C1S2 + Amp and C1S3 + Amp, respectively. Notably, the ampicillin-loaded chitosan/starch nanoparticles exhibit higher and more stable antibacterial activity against *E. coli* compared to both free ampicillin and ampicillin-loaded starch nanoparticles. In conclusion, chitosan/starch nanocomposites present stable and promising materials for drug delivery applications in the pharmaceutical field.

## Figures and Tables

**Table 1:** Characterization of chitosan/starch nanocomposites and chitosan/starch nanocomposites loaded with ampicillin.

Samples Data	C100	C1S1	C1S2	C1S3	S100	C100 + Amp	C1S1 + Amp	C1S2 + Amp	C1S3 + Amp	S100 + Amp
Size distribution (nm)	200–1300	150–600	100–500	100–400	70–400	250–1500	250–900	150–900	120–600	180–500
Mean Size (nm)	816.9 ± 24.5	372.4 ± 17.4	281.0 ± 12.3	190.0 ± 2.87	133.2 ± 4.32	1020.8 ± 32.4 ^a^	583.9 ± 25.2 ^b^	566.4 ± 20.5 ^b^	418.0 ± 17.8 ^c^	312.4 ± 15.4 ^d^
Zeta potential value (mV)	67.6 ± 0.93	80.3 ± 0.95	71.4 ± 1.01	54.4 ± 1.27	−19.7 ± 0.21	64.7 ± 1.42 ^a^	63.2 ± 0.91 ^a^	61.3 ± 0.97 ^a^	37.3 ± 1.34 ^b^	−6.3 ± 0.29 ^c^
Recovery yield (%)	75.4 ± 0.35	75.1 ± 0.06	75.0 ± 0.08	75.1 ± 0.14	75.2 ± 1.78	72.8 ± 0.34	72.5 ± 0.95	71.1 ± 1.75	72.1 ± 0.96	72.0 ± 1.45
Loading efficiency (%)	-	-	-	-	-	65.8 ± 2.87 ^b^	75.5 ± 3.21 ^a^	77.3 ± 2.97 ^a^	75.3 ± 3.15 ^a^	59.9 ± 3.68 ^c^

Data in the table are presented as mean ± SD of three replicates. Superscripts a, b, c, and d in the rows with different letters indicate significant differences (*p* < 0.05).

## Data Availability

All the data for this article can be found within the article itself.

## Supplementary Materials

The following supporting information can be downloaded at https://www.mdpi.com/article/10.3390/polym16182647/s1, Figure S1. Linear regression equation of ampicillin; Figure S2. HPLC profile of Ampicillin sodium standard; Figure S3. HPLC profile of C1S2+Amp samples at 1 h; Figure S4. HPLC profile of C1S2+Amp sample at 2 h; Figure S5. HPLC profile of C1S2+Amp sample at 4 h; Figure S6. HPLC profile of C1S2+Amp sample at 6 h; Figure S7. HPLC profile of C1S2+Amp sample at 12 h; Figure S8. HPLC profile of C1S2+Amp sample at 24 h; Figure S9: Zeta potential values of chitosan/starch nanocomposites loaded with ampicillin.

## References

[1] Aslam B., Wang W., Arshad M.I., Khurshid M., Muzammil S., Rasool M.H., Nisar M.A., Alvi R.F., Aslam M.A., Qamar M.U. (2018). Antibiotic resistance: A rundown of a global crisis. Infect. Drug Resist..

[2] WHO (2018). High Levels of Antibiotic Resistance Found Worldwide, New Data Shows. WHO, News Release. https://www.who.int/home/29-01-2018-high-levels-of-antibiotic-resistance-found-worldwide-new-data-shows.

[3] Li M., Liu Q., Teng Y. (2019). The resistance mechanism of *E. coli* induced by ampicillin in laboratory. Infect. Drug Resist..

[4] Vranic S.M., Uzunovic A. (2016). Antimicrobial Resistance of *E. coli* strains isolated from urine at outpatient population: A single laboratory experience. Mater. Socio Medica.

[5] Nwobodo D.C., Ugwu M.C., Anie C.O., Al-Ouqaili M.T.S., Ikem J.C., Chigozie U.V., Saki M. (2022). Antibiotic resistance: The challenges and some emerging strategies for tackling a global menace. J. Clin. Lab. Anal..

[6] Eleraky N.E., Allam A., Hassan S.B., Omar M.M. (2020). Nanomedicine fight against antibacterial resistance: An overview of the recent pharmaceutical innovations. Pharmaceutics.

[7] Ssekatawa K., Byarugaba D.K., Kato C.D., Ejobi F., Tweyongere R., Lubwama M., Kỉabira J.B., Wampande E.M. (2020). Nanotechnological solutions for controlling transmission and emergence of antimicrobial-resistant bacteria, future prospects, and challenges: A systematic review. J. Nanoparticle Res..

[8] Montero N., Alhajj M.J., Mariana Sierra M., Oñate-Garzon J., Yarce C.J., Salamanca C.H. (2020). Development of polyelectrolyte complex nanoparticles-PECNs loaded with ampicillin by means of polyelectrolyte complexation and ultra-high pressure homogenization (UHPH). Polymers.

[9] Ke C.L., Deng F.S., Chuang C.Y., Lin C.H. (2021). Antimicrobial actions and applications of chitosan. Polymers.

[10] Nguyen V.T., Nguyen T.T.H., Wang S.L., Vo T.T.H., Nguyen A.D. (2017). Preparation of chitosan nanoparticles by TPP ionic gelation combined with spray drying, and the antibacterial activity of chitosan nanoparticles and a chitosan nanoparticle–amoxicillin complex. Res. Chem. Intermed..

[11] Rozman N.A.S., Tong W.Y., Leong C.R., Tan W.N., Hasanolbasori M.A., Abdullah S.Z. (2019). Potential antimicrobial applications of chitosan nanoparticles (ChNP). J. Microbiol. Biotechnol..

[12] La T.K.N., Phung M.L., Wang S.L., Nguyen A.D. (2014). Preparation of chitosan nanoparticles by spray drying, and their antibacterial activity. Res. Chem. Intermed..

[13] Banoub N.G., Saleh S.E., Helal H.S., Aboshanab K.M. (2021). Antibiotics combinations and chitosan nanoparticles for combating multidrug resistance *Acinetobacter baumannii*. Infect. Drug Resist..

[14] Deng Z., Wang T., Chen X., Liu Y. (2020). Applications of chitosan-based biomaterials: A focus on dependent antimicrobial properties. Mar. Life Sci. Technol..

[15] Costa E.M., Silva S., Veiga M., Tavaria F.K., Pintado M.M. (2018). Exploring chitosan nanoparticles as effective inhibitors of antibiotic resistant skin microorganisms—From in vitro to ex vitro testing. Carbohydr. Res..

[16] Ibrahim H.M., El-Bisi M.K., Taha G.M., El-Alfy A.E. (2020). Preparation of biocompatible chitosan nanoparticles loaded by tetracycline, gentamycin and ciprofloxacin as novel drug delivery system for improvement the antibacterial properties of cellulose based fabrics. Int. J. Biol. Macromol..

[17] Sohail R., Abbas S.R. (2020). Evaluation of amygdalin-loaded alginate-chitosan nanoparticles as biocompatible drug delivery carriers for anticancerous efficacy. Int. J. Biol. Macromol..

[18] Ehyaeirad N., Babolanimogadam N., Dadkhah M., Shirmard L.R. (2024). Polylactic acid films incorporated with nanochitosan, nanocellulose, and Ajwain essential oil: Synthesis, characterizations, with in-vitro and in-vivo antimicrobial properties for infected wound healing. Carbohydr. Polym. Technol. Appl..

[19] Marta H., Rizki D.I., Mardawati E., Djali M., Mohammad M., Cahyana Y. (2023). Starch nanoparticles: Preparation, properties and applications. Polymers.

[20] Gheorghita R., Anchidin-Norocel L., Filip R., Dimian M., Covasa M. (2021). Applications of biopolymers for drugs and probiotics delivery. Polymers.

[21] Hemamalini T., Giri Dev V.R. (2018). Comprehensive review on electro spinning of starch polymer for biomedical applications. Int. J. Biol. Macromol..

[22] Wang B., Sui J., Yu B., Yuan C., Guo L., Abd El-Aty A.M., Cui B. (2021). Physicochemical properties and antibacterial activity of corn starch-based films incorporated with *Zanthoxylum bungeanum* essential oil. Carbohydr. Polym..

[23] Alwaan I.M., Ahmed M., Al-Kelaby K.K.A., Allebban Z.S.M. (2018). Starch-chitosan modified blend as long term controlled drug release for cancer therapy. Pak. J. Biotechnol..

[24] Balmayor E.R., Tuzlakoglu K., Azevedo H.S., Reis R.L. (2009). Preparation and characterization of starch-poly-ε-caprolactone microparticles incorporating bioactive agents for drug delivery and tissue engineering applications. Acta Biomater..

[25] Schumacher I., Margalit R. (1997). Liposome-encapsulated ampicillin: Physicochemical and antibacterial properties. J. Pharm. Sci..

[26] Asadpoor M., Varasteh S., Pieters R.J., Folkerts G., Braber S. (2021). Differential effects of oligosaccharides on the effectiveness of ampicillin against *Escherichia coli* in vitro. PharmaNutrition.

[27] Wan Mat Khalir W.K.A., Shameli K., Miyake M., Nor A.O. (2018). Efficient one-pot biosynthesis of silver nanoparticles using *Entada spiralis* stem powder extraction. Res. Chem. Intermed..

[28] Hussein-Al-Ali S.H., El Zowalaty M.E., Hussein M.Z., Geilich B.M., Webster T.J. (2014). Synthesis, characterization, and antimicrobial activity of an ampicillin-conjugated magnetic nanoantibiotic for medical applications. Int. J. Nanomed..

[29] Abdel-Hakeem M.A., Maksoud A.I.A., Aladhadh M.A., Almuryif K.A., Elsanhoty R.M., Elebeedy D. (2022). Gentamicin–Ascorbic acid encapsulated in chitosan nanoparticles improved in vitro antimicrobial activity and minimized cytotoxicity. Antibiotics.

[30] Abdelmalek I., Svahn I., Mesli S., Simonneaux G., Mesli A. (2014). Formulation, evaluation and microbiological activity of ampicillin and amoxicillin microspheres. J. Mater. Environ. Sci..

[31] Ibrahim H.M., El-Bisi M.K., Taha G.M., El-Alfy E.A. (2015). Chitosan nanoparticles loaded antibiotics as drug delivery biomaterial. J. Appl. Pharm. Sci..

[32] Fan P., Ma Z., Partow A.J., Kim M., Grace M., Shoemaker G.M., Tan R., Tong Z., Nelson C.D., Jang Y. (2022). A novel combination therapy for multidrug resistant pathogens using chitosan nanoparticles loaded with β-lactam antibiotics and β-lactamase inhibitors. Int. J. Biol. Macromol..

[33] Murei A., Ayinde W.B., Gitari M.W., Samie A. (2020). Functionalization and antimicrobial evaluation of ampicillin, penicillin and vancomycin with *Pyrenacantha grandiflora* Baill and silver nanoparticles. Sci. Rep..

[34] Nairi V., Medda L., Monduzzi M., Salis A. (2017). Adsorption and release of ampicillin antibiotic from ordered mesoporous silica. J. Colloid Interface Sci..

[35] Abdullah A.H.D., Chalimah S., Primadona I., Hanantyo M.H.G. (2018). Physical and chemical properties of corn, cassava, and potato starch. IOP Conf. Ser. Earth Environ. Sci..

[36] Hong T., Yin J.Y., Nie S.P., Xie M.Y. (2021). Applications of infrared spectroscopy in polysaccharide structural analysis: Progress, challenge and perspective. Food Chem. X.

[37] Lustriane C., Dwivany F.M., Suendo V., Reza M. (2018). Effect of chitosan and chitosan-nanoparticles on post-harvest quality of banana fruits. J. Plant Biotechnol..

[38] Queiroz M.F., Melo K.R.T., Sabry D.A., Sassaki G.L., Rocha H.A.O. (2015). Does the use of chitosan contribute to oxalate kidney stone formation. Mar. Drugs.

[39] Duceac L.D., Calin G., Eva L., Marcu C., Goroftei E.R.B., Dabija M.G., Mitrea G., Luca A.C., Hanganu E., Gutu C. (2020). Third-generation cephalosporin-loaded chitosan used to limit microorganisms resistance. Materials.

[40] Najafi S.H.M., Baghaie M., Ashori A. (2016). Preparation and characterization of acetylated starch nanoparticles as drug carrier: Ciprofloxacin as a model. Int. J. Biol. Macromol..

[41] Sivamaruthi B.S., Nallasamy P.K., Suganthy N., Kesika P., Chaiyasut C. (2022). Pharmaceutical and biomedical applications of starch-based drug delivery system: A review. J. Drug Deliv. Sci. Technol..

[42] Fazeli M., Lipponen J. (2022). Developing self-assembled starch nanoparticles in starch nanocomposite films. ACS Omega.

