# Supplementary material for: Preparation and Characterization of Chitosan/Starch Nanocomposites Loaded with Ampicillin to Enhance Antibacterial Activity against Escherichia coli
Source: Polymers (Basel). 2024 Sep 19;16(18):2647. doi: 10.3390/polym16182647 (PMC11435967; doi:10.3390/polym16182647)
Supplement: Supplementary file 1 [file polymers-16-02647-s001.zip › polymers-3191060-supplementary.pdf]

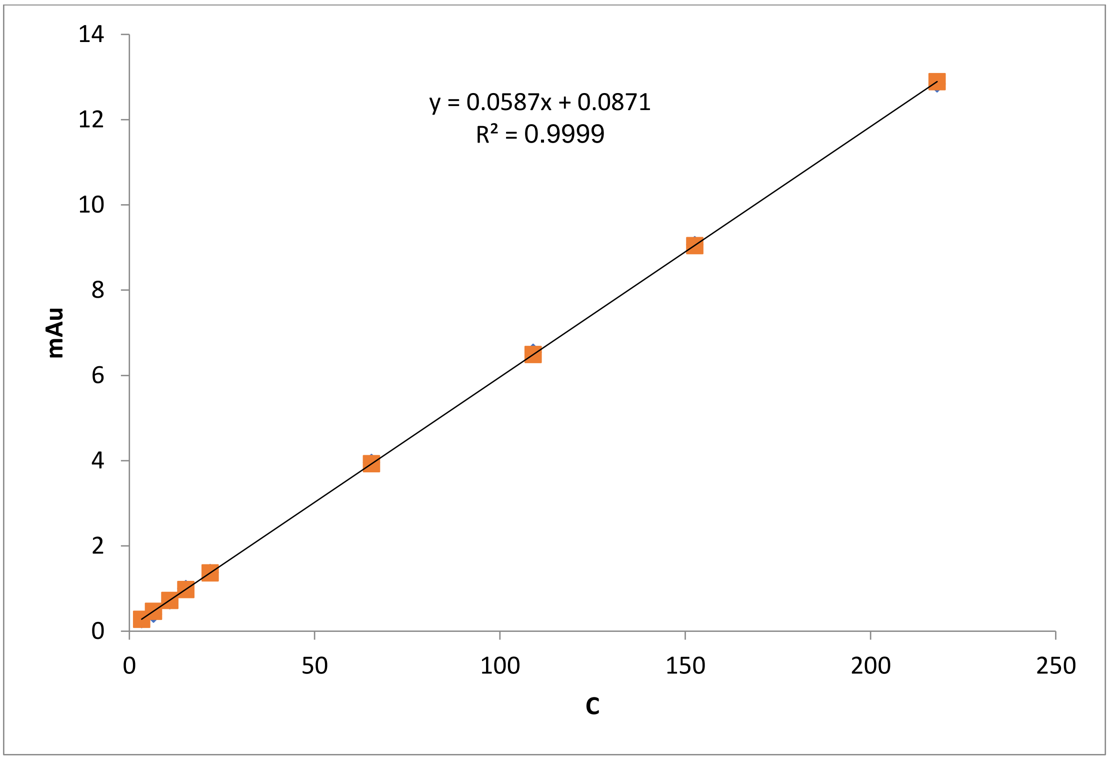

Figure S1. Linear regression equation of ampicillin

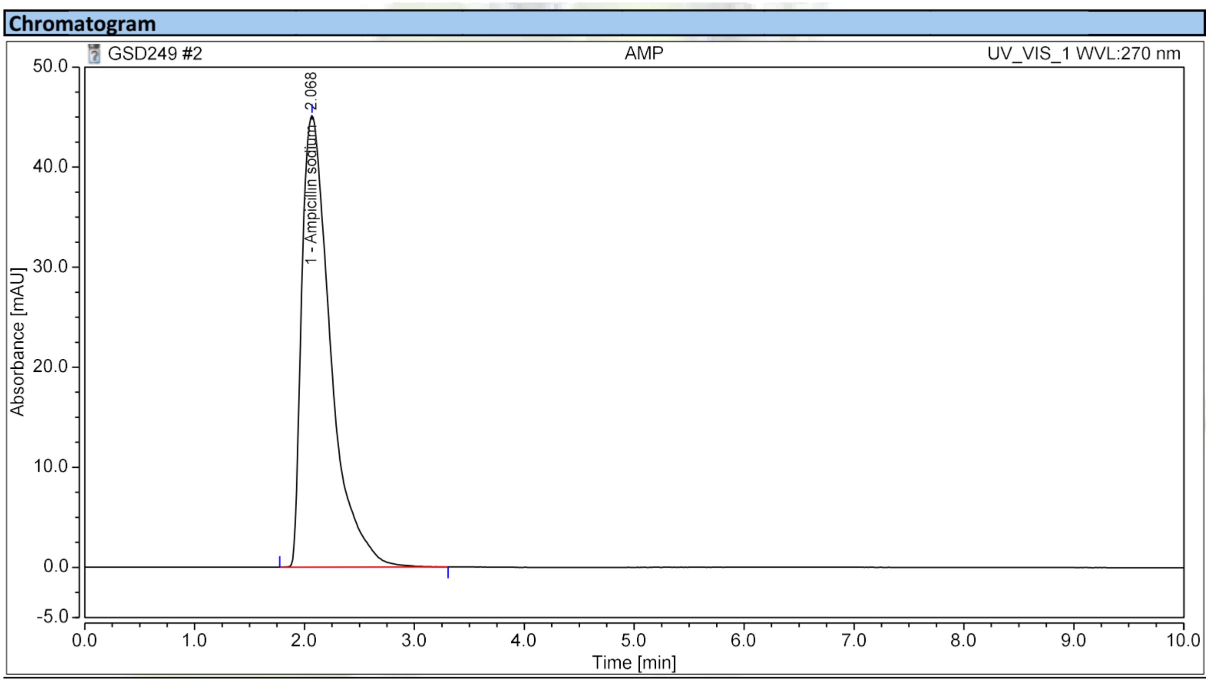

Figure S2. HPLC profile of Ampicillin sodium standard

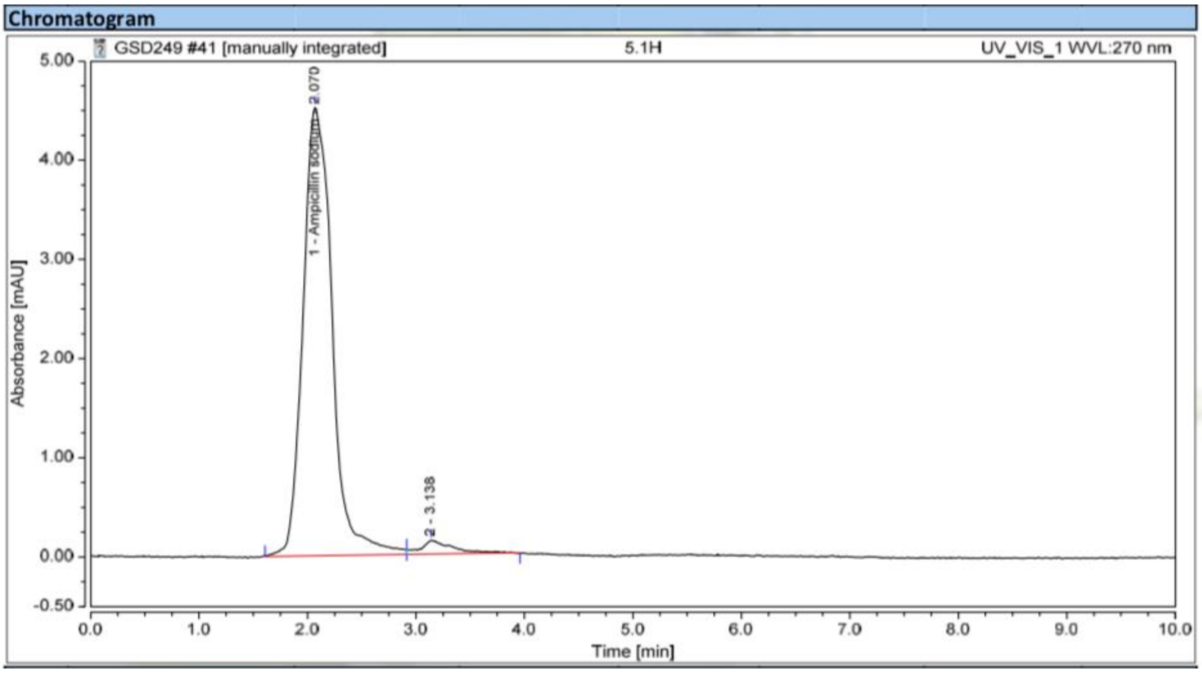

Figure S3. HPLC profile of C1S2+Amp samples at 1h

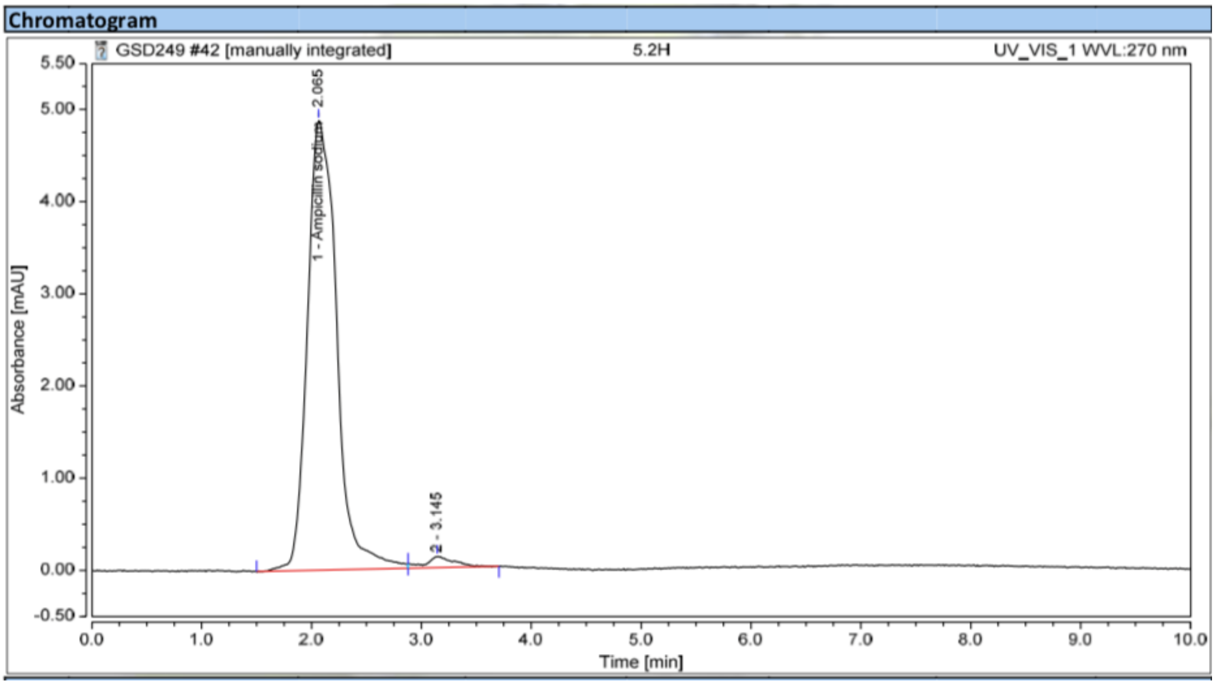

Figure S4. HPLC profile of C1S2+Amp sample at 2h

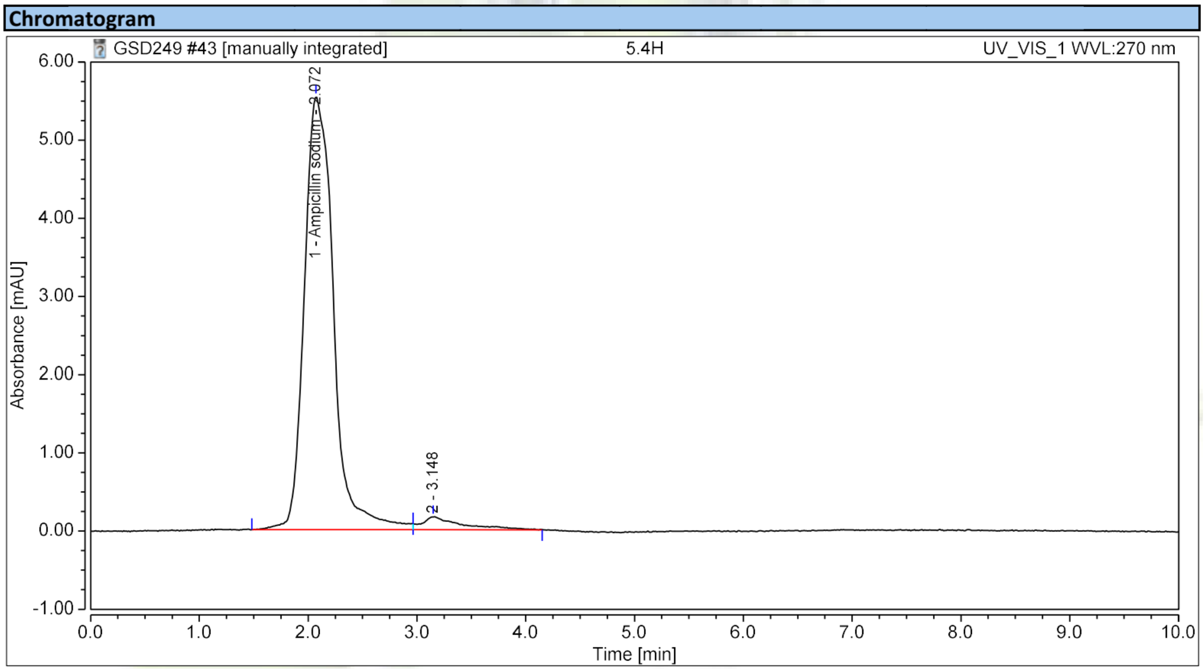

Figure S5. HPLC profile of C1S2+Amp sample at 4h

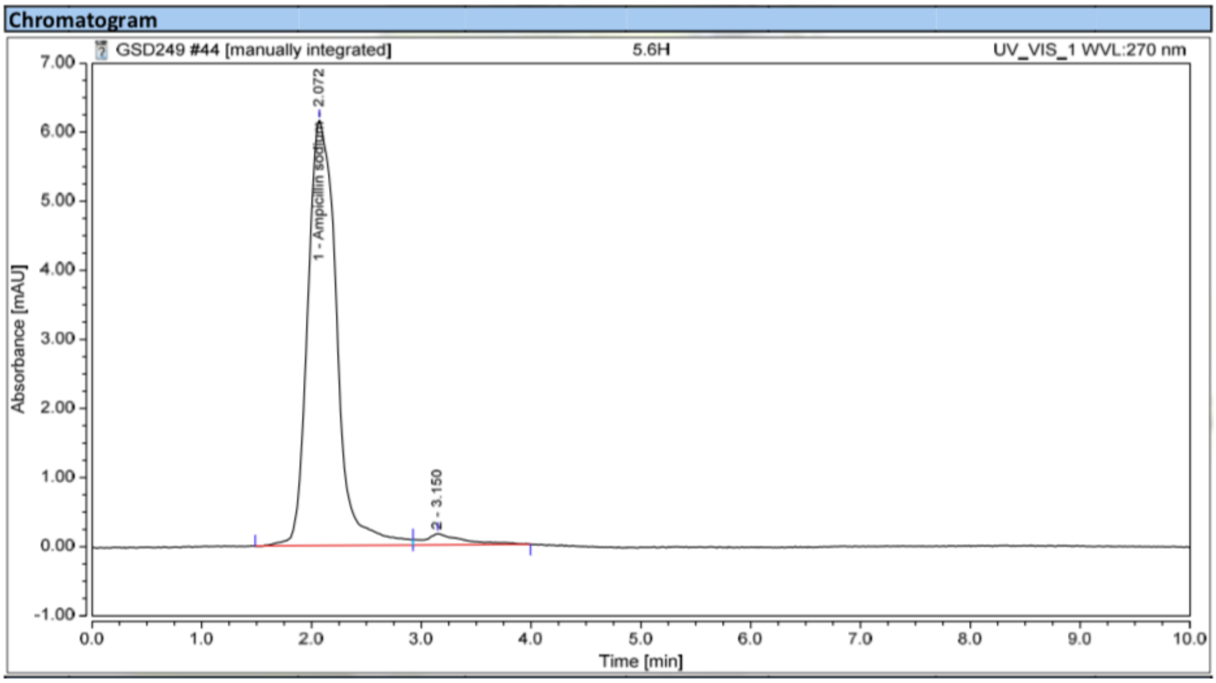

Figure S6. HPLC profile of C1S2+Amp sample at 6h

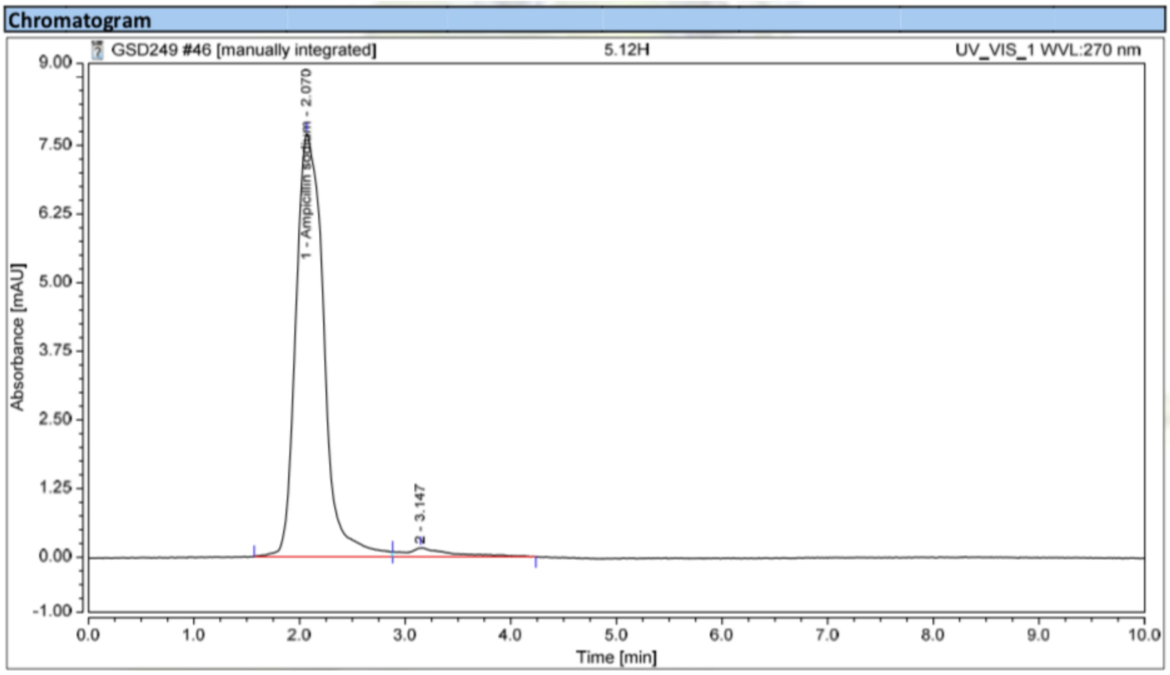

Figure S7. HPLC profile of C1S2+Amp sample at 12h

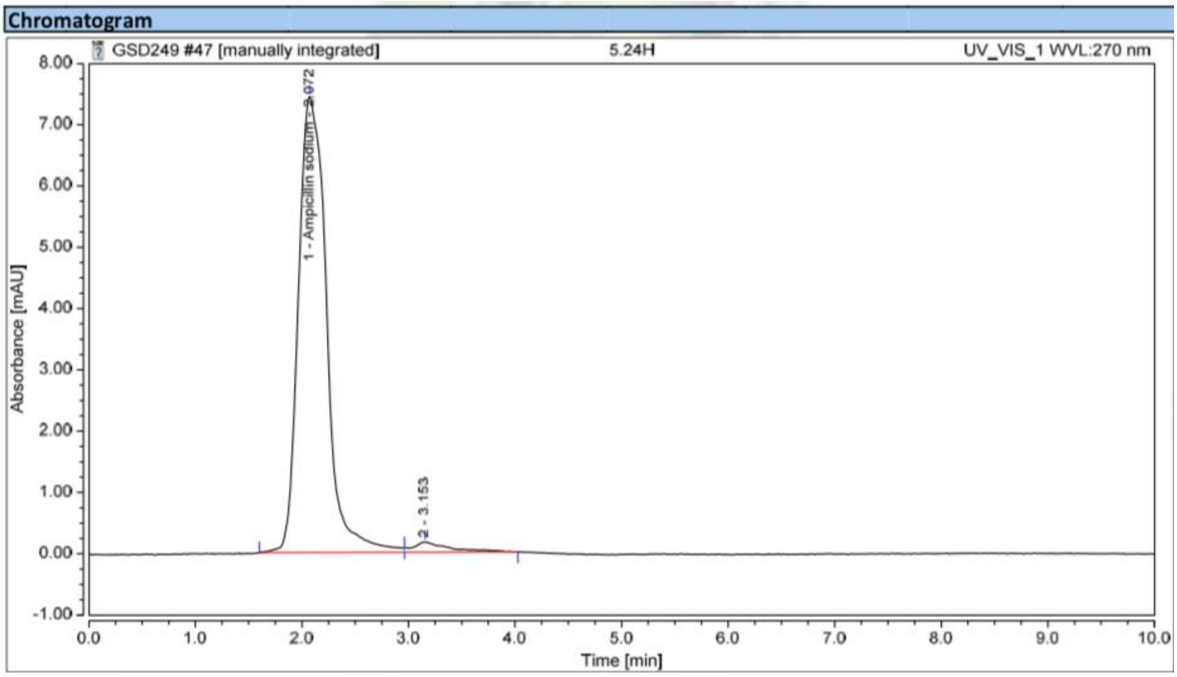

Figure S8. HPLC profile of C1S2+Amp sample at 24 h

Supplement documents Figure S9 Zeta potential

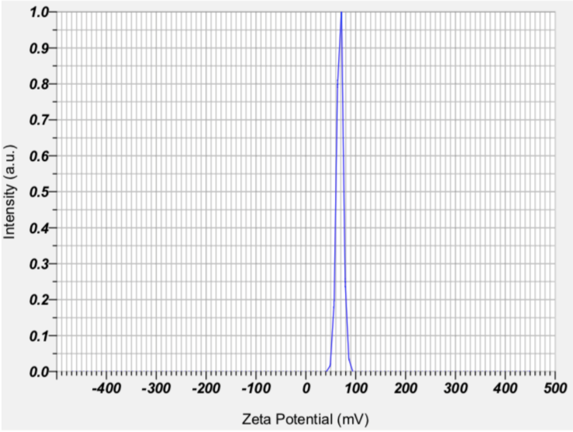

a) C100

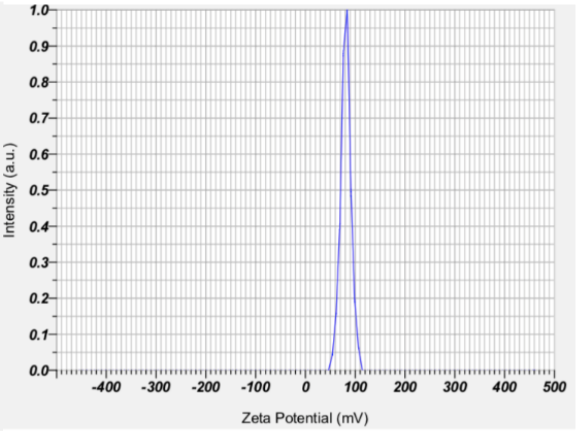

b) C1S1

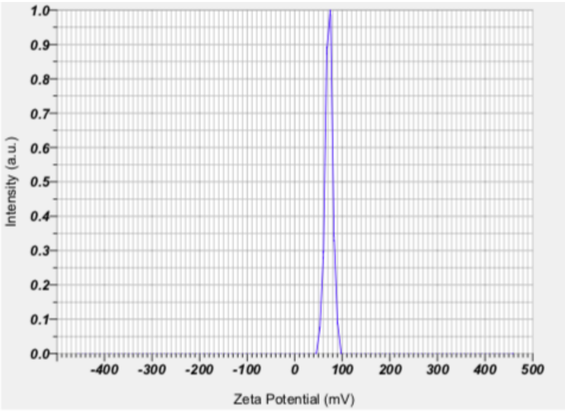

c) C1S2

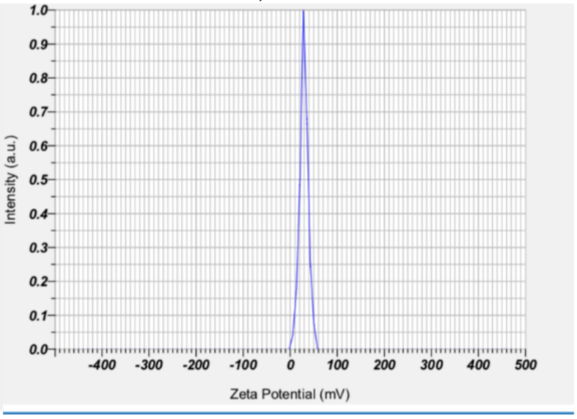

d) C1S3

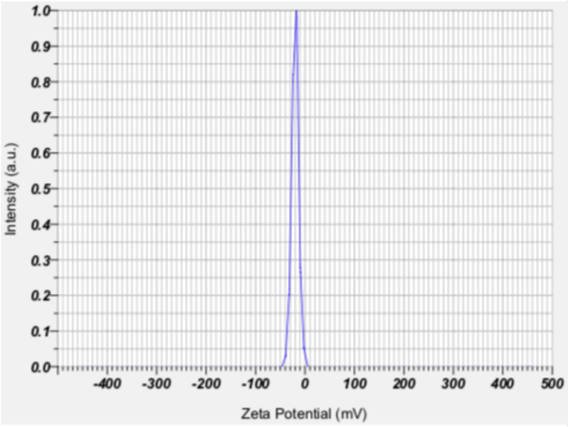

e) S100

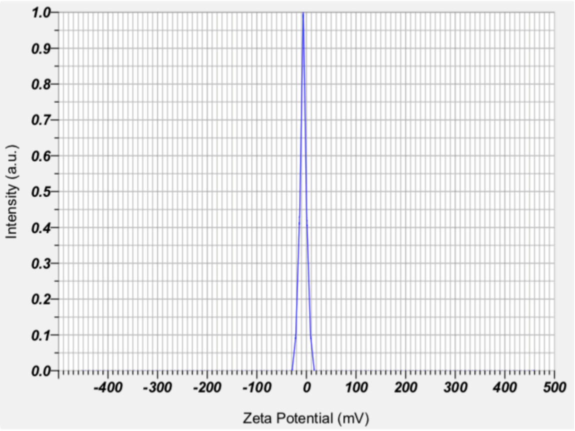

f) S100 + Amp

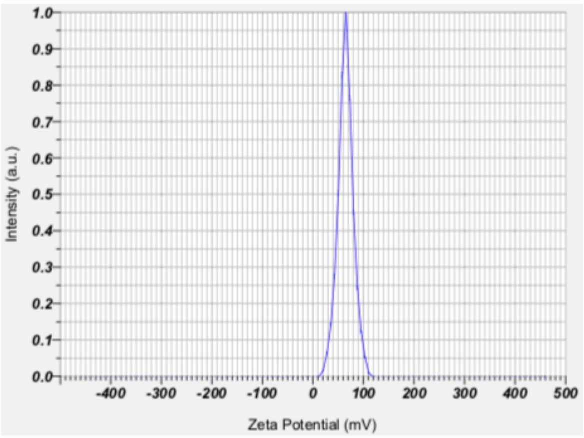

f) C100+Amp

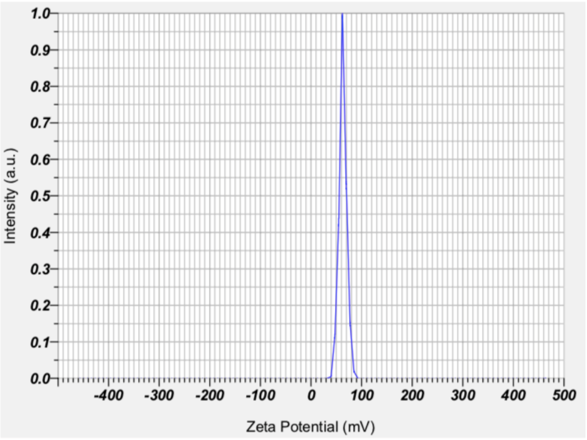

g) C1S1+Amp

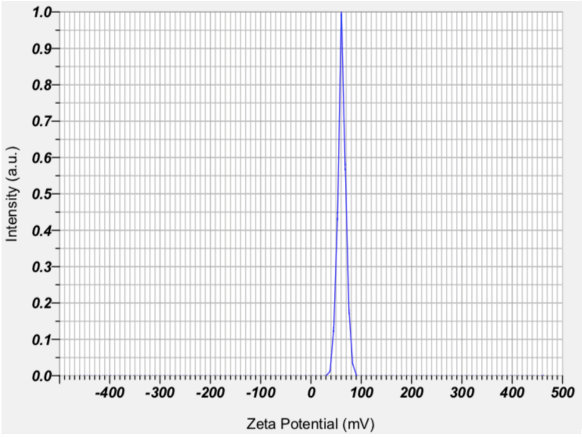

h) C1S2+Amp

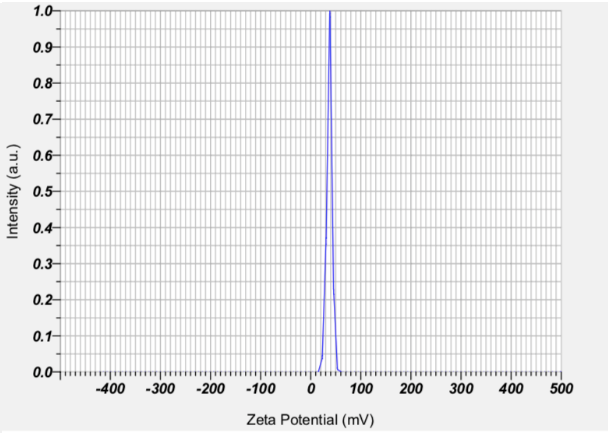

i) C1S3+Amp

Figure S9. Zeta potential values of chitosan/starch nanocomposites and chitosan/ starch nanocomposites loaded ampicillin
